# Renshen Yangrong decoction for secondary malaise and fatigue: network pharmacology and Mendelian randomization study

**DOI:** 10.3389/fnut.2024.1404123

**Published:** 2024-06-20

**Authors:** Fanghan Wang, Liping Zhu, Haiyan Cui, Shanchun Guo, Jingliang Wu, Aixiang Li, Zhiqiang Wang

**Affiliations:** ^1^Department of Medical Oncology, The Fourth People’s Hospital of Zibo, Zibo, China; ^2^Department of Medical Oncology, Shouguang Hospital of Traditional Chinese Medicine, Shouguang, China; ^3^Department of Pathology, The Fourth People’s Hospital of Zibo, Zibo, China; ^4^RCMI Cancer Research Center, Xavier University of Louisiana, New Orleans, LA, United States; ^5^Medical School, Weifang University of Science and Technology, Shouguang, China; ^6^Department of Urology, Shouguang Hospital of Traditional Chinese Medicine, Shouguang, China

**Keywords:** Renshen Yangrong decoction, secondary malaise and fatigue, network pharmacology, Mendelian randomization, molecular docking

## Abstract

**Background:**

Renshen Yangrong decoction (RSYRD) has been shown therapeutic effects on secondary malaise and fatigue (SMF). However, to date, its bioactive ingredients and potential targets remain unclear.

**Purpose:**

The purpose of this study is to assess the potential ingredients and targets of RSYRD on SMF through a comprehensive strategy integrating network pharmacology, Mendelian randomization as well as molecular docking verification.

**Methods:**

Search for potential active ingredients and corresponding protein targets of RSYRD on TCMSP and BATMAN-TCM for network pharmacology analysis. Mendelian randomization (MR) was performed to find therapeutic targets for SMF. The eQTLGen Consortium (sample sizes: 31,684) provided data on cis-expression quantitative trait loci (cis-eQTL, exposure). The summary data on SMF (outcome) from genome-wide association studies (GWAS) were gathered from the MRC-IEU Consortium (sample sizes: 463,010). We built a target interaction network between the probable active ingredient targets of RSYRD and the therapeutic targets of SMF. We next used drug prediction and molecular docking to confirm the therapeutic value of the therapeutic targets.

**Results:**

In RSYRD, network pharmacology investigations revealed 193 possible active compounds and 234 associated protein targets. The genetically predicted amounts of 176 proteins were related to SMF risk in the MR analysis. Thirty-seven overlapping targets for RSYRD in treating SMF, among which six (NOS3, GAA, IMPA1, P4HTM, RB1, and SLC16A1) were prioritized with the most convincing evidence. Finally, the 14 active ingredients of RSYRD were identified as potential drug molecules. The strong affinity between active components and putative protein targets was established by molecular docking.

**Conclusion:**

This study revealed several active components and possible RSYRD protein targets for the therapy of SMF and provided novel insights into the feasibility of using Mendelian randomization for causal inference between Chinese medical formula and disease.

## 1 Introduction

Fatigue, in medical terminology, is defined as a notable deficiency in both physical and mental energy that is seen by the subject or caregiver as hindering normal or desirable activities. It can be categorized as either physiological, secondary, or chronic (such as in the case of Myalgic Encephalomyelitis) (1, 2). Secondary malaise and fatigue (SMF) is caused by disease-related symptoms such as depression, decreased activity, sleep disturbance, anemia, hypothyroidism, malnutrition, and infections rather than the disease itself (3, 4). Furthermore, the therapy of the condition, or medication in general, might lead to secondary weariness, particularly when numerous pharmacotherapies (“polypharmacy”) are used (5). Currently, treatment mainly targets potential diseases. For example, pulmonary rehabilitation helps the respiratory symptoms of chronic obstructive pulmonary disease and also fatigue (6). Exercise therapy and psychological therapies, notably cognitive behavior therapy, offer some evidence of success in the treatment of fatigue caused by cancer, inflammatory illnesses (e.g., inflammatory bowel disease, rheumatoid arthritis) (7–9), and neurological disorders (for example, multiple sclerosis, myasthenia gravis) (10, 11). Exercise may improve fatigue and function related to fibromyalgia (12, 13). Tai chi may be superior to aerobic exercise for those with fibromyalgia (14). Massage and acupuncture may help manage cancer-related fatigue (15, 16). Understanding SMF involves recognizing it as a symptom rather than a standalone condition. Alongside treating the root cause, patients may benefit from general strategies to reduce fatigue, such as regular physical activity, balanced nutrition, stress management techniques, and pacing daily activities to avoid overexertion (17). Effectively managing SMF requires a comprehensive, individualized approach that targets the specific underlying cause while also incorporating general strategies to improve overall energy levels and well-being. There is no evidence that pharmacologic treatment targeting fatigue (e.g., modafinil, methylphenidate) helps manage fatigue related to most chronic diseases (18, 19). Thus, further efforts are still urgently needed to develop novel strategies to prevent this secondary malaise and fatigue.

Zhubing Yuanhou Lun, a well-known tract about the etiology and symptoms of disease produced during the Sui Dynasty, contains almost 70 different types of symptoms in its chapter on “consumptive disease.” The symptoms can be divided into two categories: psychological symptoms like depression, anxiety, restlessness, and so on, and somatic symptoms like fatigue, a somatic sense of heaviness, and somatic pain (joint and muscle pain), among others. Traditional Chinese Medicine (TCM) categorizes SMF as a “consumptive disease.” Patients may have poor blood and Qi due to extended exposure to pollutants, overwork, emotional distress, or incorrect food. This can lead to renal function impairment and five-organ failure, ultimately culminating in SMF. Additionally, blood and Qi impairment are aggravated by chemotherapy, radiotherapy, and surgery, which exacerbates fatigue symptoms. Thus, Qi/blood deficiency and dysfunction of the five organs are implicated within the aetiology of SMF in TCM. To make up for the shortage, SMF is primarily treated by tonifying the body and strengthening its resistance. RSYRD (Ninjin’yoeito in Japanese) was first documented in Taiping Huimin Heji Jufang, a classic ancient Chinese medical text first published in 1078 during the Song Dynasty. Its efficacy in treating anemia (20), fatigue (21–27), general malaise brought on by chemotherapy and malignant tumours (25, 27), as well as psychological conditions like anxiety and depression and the lethargic and apathetic symptoms of Parkinson’s and Alzheimer’s diseases (28–31), has been demonstrated in earlier clinical and preclinical research. Despite extensive research, due to the diverse composition and functions of Chinese herbal medicines, the specific mechanisms by which they exert their corresponding effects are difficult to explore through basic experiments, limiting the widespread clinical application of RSYRD worldwide.

Incorporating genetics into the production of medications is a highly effective approach to expedite this process, as medicines based on genetics have a far higher likelihood of success in clinical trials (32, 33). Druggable genes that encode proteins have now been identified as possible targets for pharmaceuticals, small compounds, and monoclonal antibodies (34, 35). GWAS can effectively identify single nucleotide polymorphisms (SNPs) related with disease risk, but it cannot reliably identify causal genes and drive therapeutic development (36, 37). The integration of network pharmacology and Mendelian randomization provides a practical approach to exploring the pharmacology mechanism of Chinese medical formulae and evaluating the pharmacological modulation of the gene target. Drug target Mendelian randomization is an application of MR in drug target validation. It employs genetic instruments linked to drug target genes to determine whether there is a link between the medication target and disease outcome (34, 38). Network pharmacology can systematically reveal the active ingredients in Chinese herbal medicines and predict the relationships between drug compounds and protein targets (39). Molecular docking could be used to validate the interaction between active components and key therapeutic targets (40). Finally, our findings provide vital insights into new theoretical support for the therapeutic therapy of SMF. We provide informative advice for the development of more effective and focused therapeutic modalities by integrating drug target Mendelian randomization, network pharmacology, molecular docking, and drug prediction.

## 2 Materials and methods

### 2.1 Composition of Renshen Yangrong decoction

RSYRD is a decoction of 12 botanicals after water extraction, which includes Renshen (*Panax Ginseng C. A. Mey.*), Baizhu (*Atractylodes Macrocephala Koidz.*), Chenpi (*Citrus Reticulata*), Gancao (*licorice*), Fuling (*Poria Cocos (Schw.) Wolf.*), Huangqi (*Hedysarum Multijugum Maxim.*), Baishao (*Paeoniae Radix Alba*), Danggui (*Angelicae Sinensis Radix*), Shudihuang (*Rehmanniae Radix Praeparata*), Wuweizi (*Schisandrae Chinensis Fructus*), Rougui (*Cinnanmomi Cortex*), Yuanzhi (*Polygala tenuifolia Willd.*), and they all possess medicinal values. The drug names refer to the 2020 edition of the Chinese Pharmacopeia and the plant classification refers to the MPNS database.^1^

### 2.2 Screening the active ingredients and targets of RSYRD

The active components of RSYRD, as well as their related targets, were collected from the Traditional Chinese Medicine Systems Pharmacology (TCMSP) Database and Analysis Platform^2^ by using “Renshen,” “Baizhu,” “Fuling,” “Gancao,” “Chenpi,” “Huangqi,” “Danggui,” “Baishao,” “Shudihuang” and “Wuweizi” as keywords. However, the chemical components of “Yuanzhi” and “Rougui” were taken through the BATMAN-TCM database^3^ (41) and the obtained compounds were then further screened by TCSMP. The TCMSP database advised screening bioactive compounds based on threshold values of drug-like characteristics (DL) ≥0.18 and oral bioavailability (OB) ≥30% (42). The UniProt database^4^ was used to standardize all target names.

### 2.3 Exposure and outcome dataset

The eQTLGen consortium^5^ provided the whole set of cis-eQTLs data and allele frequency statistics (43). The 4,463 genes on the list of druggable genes are from a previous study (35, 44). The eQTLs analyzed in our study were restricted to SNPs positioned within a 100 kilobase (kb) range before or after the endpoint of a druggable gene. This selection criterion was based on the proximity to the gene of interest in drug development research and the potential for more direct regulation of gene expression. At last, eQTLs for 1,450 druggable genes have been identified. We used GWAS analysis of SMF from MRC-IEU Consortium as outcome variables, including genotype data of 1,245 SMF patients and 461,765 controls.^6^

### 2.4 Mendelian randomisation analysis

MRPRESSO and TwoSampleMR (version 0.5.6) were the R packages used to perform MR studies (45). Initially, we aligned the instrumental variables that target exposure-related drugs with the outcome datasets. Subsequently, we conducted analysis using several methods including MR Egger, weighted median, inverse variance weighted (IVW), simple mode, weighted mode, and MR-PRESSO. Among these approaches, the IVW method was the most commonly employed. The MR Egger and IVW techniques were used to perform the heterogeneity test (46). The *Q* value of Cochrane was used to assess the heterogeneity of genetic tools, and *p* > 0.05 indicated that there was no substantial heterogeneity. The MR Egger regression equation was used to assess the genetic tool’s horizontal pleiotropy, and *p* > 0.05 indicated that there was no horizontal pleiotropy (47). After removing the outlier with the MR-PRESSO test, the sensitivity analysis was repeated. To ensure that no SNP had a substantial impact on our results, we utilized the leave-one-out approach to delete each SNP in turn and compared the IVW method results with all variants. FDR-corrected *p* values were calculated, and FDR of <0.05 was considered significant.

### 2.5 Herb-ingredient-target network construction

A network of interactions between herbs, ingredients, and targets was generated by inputting active substances in RSYRD, common targets of these active compounds, and SMF into the Cytoscape 3.9.1 program (48). Node represents active ingredients, herbs, and targets, while edge represents the relationship between different nodes. The potential core active ingredients of RSYRD in the therapy of SMF were conjectured using the Cytoscape software’s Network Analyzer tool in conjunction with the primary active ingredients, core targets, and key herbs. Furthermore, the multi-component and multitarget synergistic effects of Chinese herbal medications were assessed in the context of SMF-related networks.

### 2.6 GO and KEGG pathway enrichment analyses

To investigate the role of identified prospective therapeutic target genes in functional characteristics and biological mechanisms, we conducted Gene Ontology (GO) and Kyoto Encyclopedia of Genes and Genomes (KEGG) pathway enrichment analysis using the “clusterProfiler” (49) and “Pathview” (50) package of R software (version 4.1.3). GO includes three terms: biological process (BP), cellular component (CC), and molecular function (MF), displaying the top 10 terms of each category, respectively. The KEGG pathway can provide metabolic pathway information. The results of all biological pathway enrichment analyses were visualized using the “ggplot2” package. The *p* < 0.05 of enriched terms were considered significantly enriched.

### 2.7 Protein–protein interaction and druggability prediction

A PPI network was constructed using the STRING database^7^ to investigate potential interactions among the identified proteins. The discovered proteins have been further analysed for possible use as therapeutic targets by searching Drug Signatures Database^8^ for drug-gene interactions (51). This database prioritised druggable targets through the integration of information gathered through text mining, expert curation, drug-gene interactions, as well as gene function. The Enrichr platform^9^ analyzed and visualized data (52).

### 2.8 Molecular docking of active ingredients with core targets

The 2D structures of all the substances were obtained from the PubChem database^10^ and stored in “SDF” format. The software Chem 3D was utilized to transform the “SDF” file format into mol2 structures, specifically for small molecule ligands. The key targets’ 3D structures were retrieved from the Protein Database (PDB)^11^ and saved as protein receptors in “PDB” format. The PyMOL program (version 2.4.0) was used to isolate the original ligands from the main target proteins. The AutoDock software (version 4.2.0) was used for importing the processed protein targets and performing hydrogenation, total charge estimation, as well as atom type setting. Protein receptors and ligands were recorded using the PDBQT format. AutoDock-Vina software (version 1.5.6) was used for molecular docking in order to provide a comprehensive score of the affinity of the receptor-ligand complexes. Heatmaps were plotted to visualize these values in R. The PyMOL software served for visualizing the docking results.

### 2.9 Identification of DEGs and clinical characteristics of key targets

**Figure 1:**
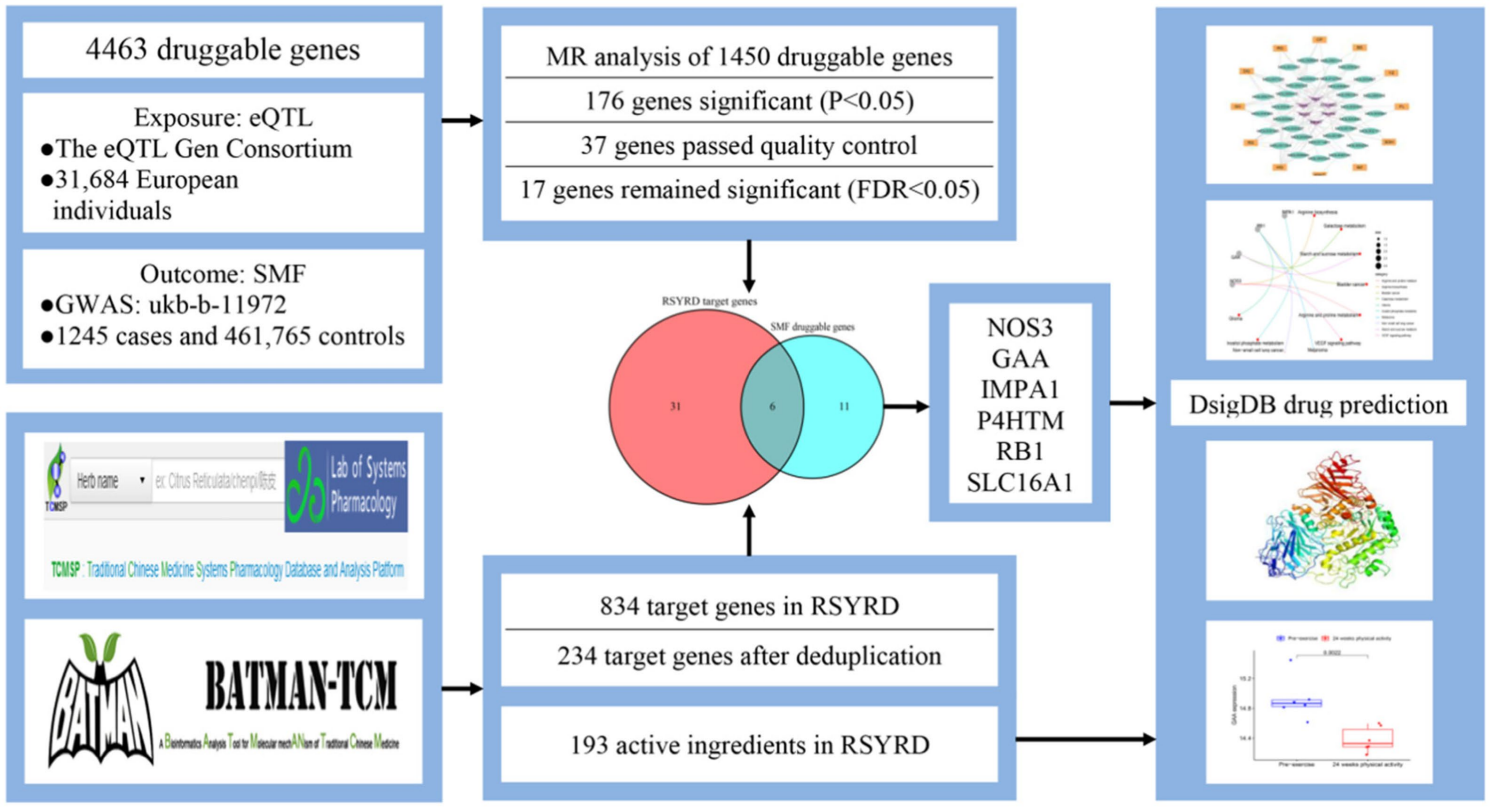
Overview of the study design.

To obtain clinical implications of potential key targets, we searched gene expression changes in Peripheral Blood Mononuclear cells (PBMC) induced by physical activity in the GEO database.^12^ The GEO dataset GSE12385 was used to validate the key genes. With |log2 fold change (FC)| >1 and *p* < 0.05 as screening criteria, differentially expressed genes (DEGs) from GSE12385 were identified utilizing “Limma” R package, where log FC >1, *p* < 0.05 was Up, log FC <−1, *p* < 0.05 was Down. The heatmap, volcano plot and box plot were conducted using “heatmap” and “ggplot2” packages of R software. Flow chart of this study was presented in Figure 1.

## 3 Results

### 3.1 Active ingredients in RSYRD and targets screening

After screening, a total of 193 active ingredients in RSYRD from TCMSP and BATMAN-TCM databases were found: 22 from Renshen, 7 from Baizhu, 15 from Fuling, 92 from Gancao, 5 from Chenpi, 20 from Huangqi, 2 from Danggui, 13 from Baishao, 2 from Shudihuang, 8 from Wuweizi, 3 from Rougui, and 4 from Yuanzhi. After normalizing the protein targets from the TCMSP database using the Uniprot database, 834 gene targets have been identified, containing 101 from Renshen, 18 from Baizhu, 19 from Fuling, 217 from Gancao, 62 from Chenpi, 196 from Huangqi, 48 from Danggui, 85 from Baishao, 28 from Shudihuang, 18 from Wuweizi, 26 from Rougui, and 16 from Yuanzhi. Following the merger, 234 distinct values remained after 600 redundant gene targets were eliminated.

### 3.2 Candidate druggable genes for SMF

**Figure 2:**
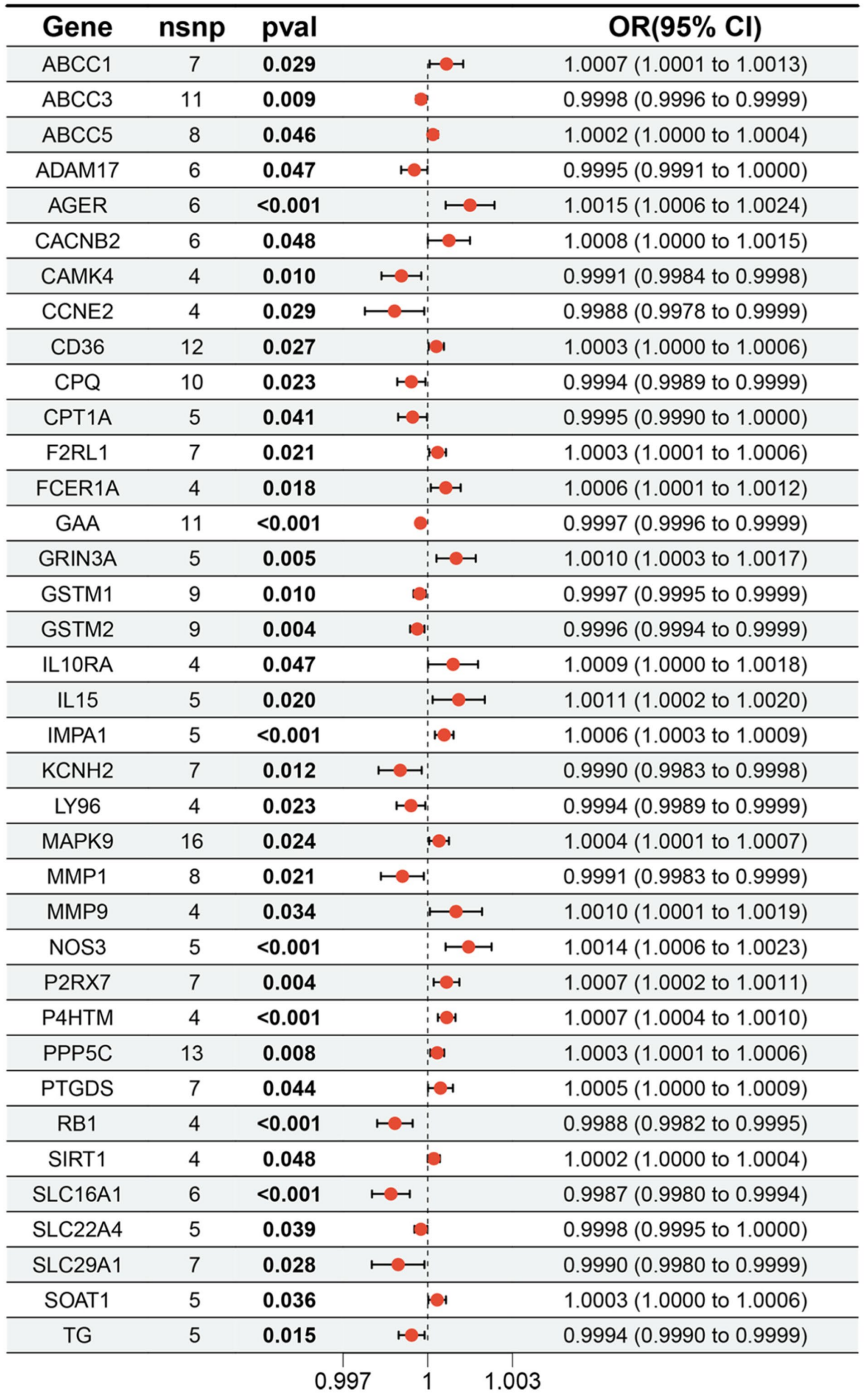
The forest plot displays the results of 37 candidate druggable genes.

**Figure 3:**
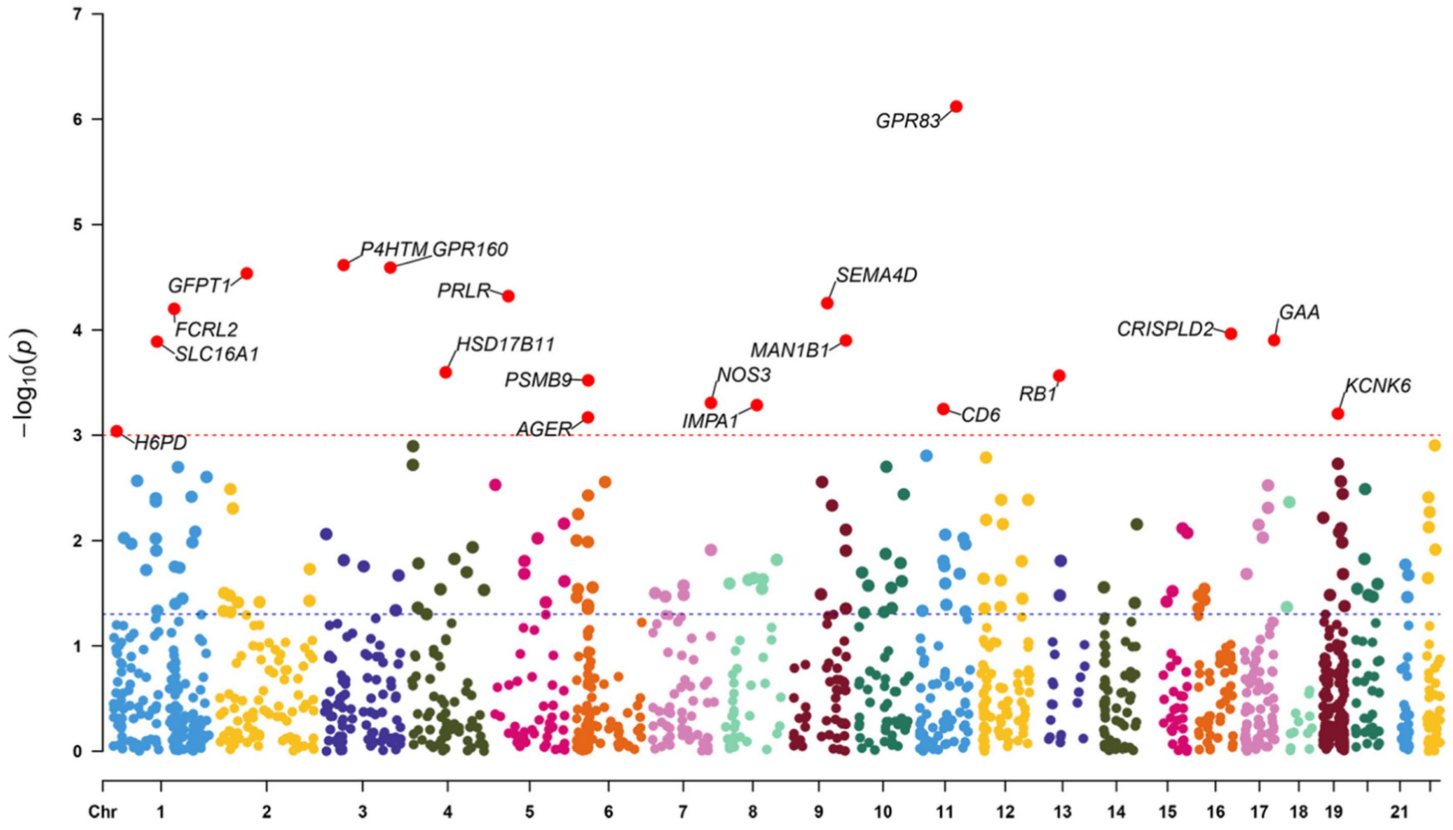
Manhattan plot of Mendelian randomization analysis. The blue line represents the nominal significant threshold of 0.05. The red line represents the false discovery rate threshold of 0.05.

**Figure 4:**
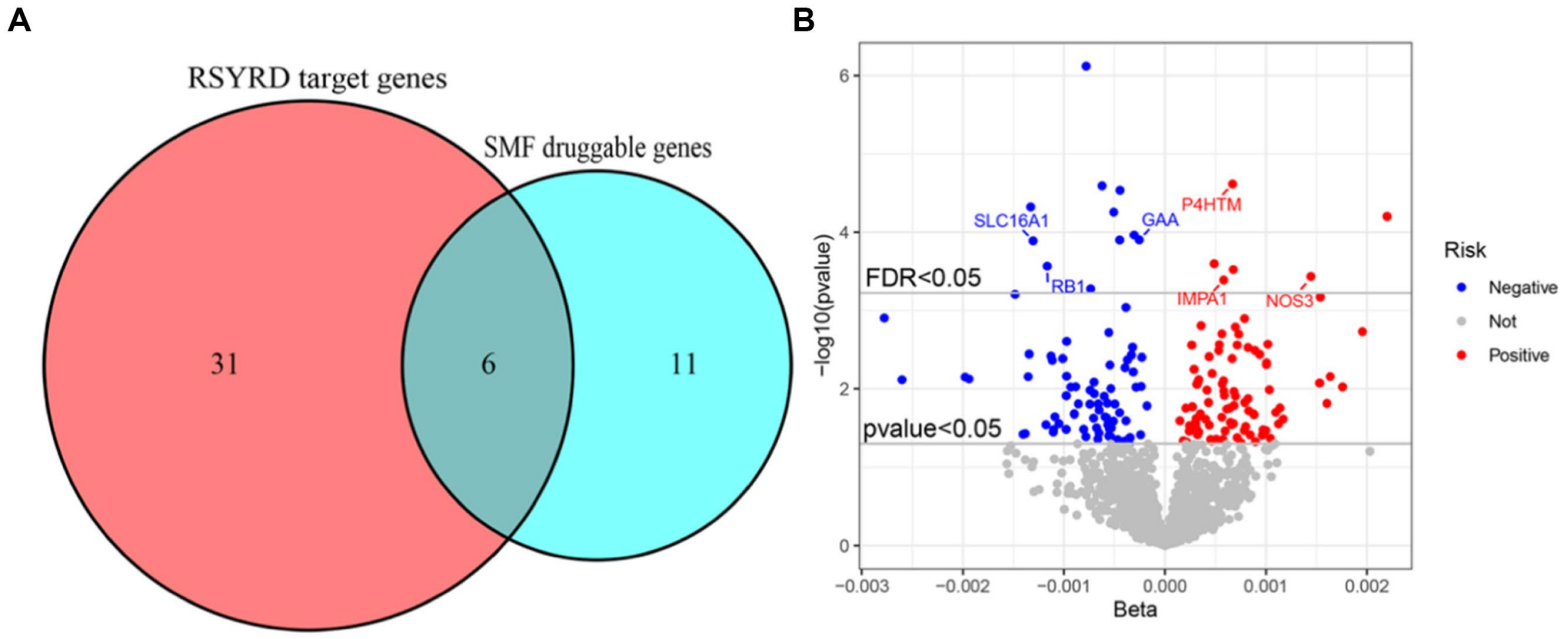
Target genes for RSYRD treatment of SMF. **(A)** Venn diagram of RSYRD target genes and SMF druggable genes. **(B)** Volcano plot showing the results of proteome-wide Mendelian randomization.

**Figure 5:**
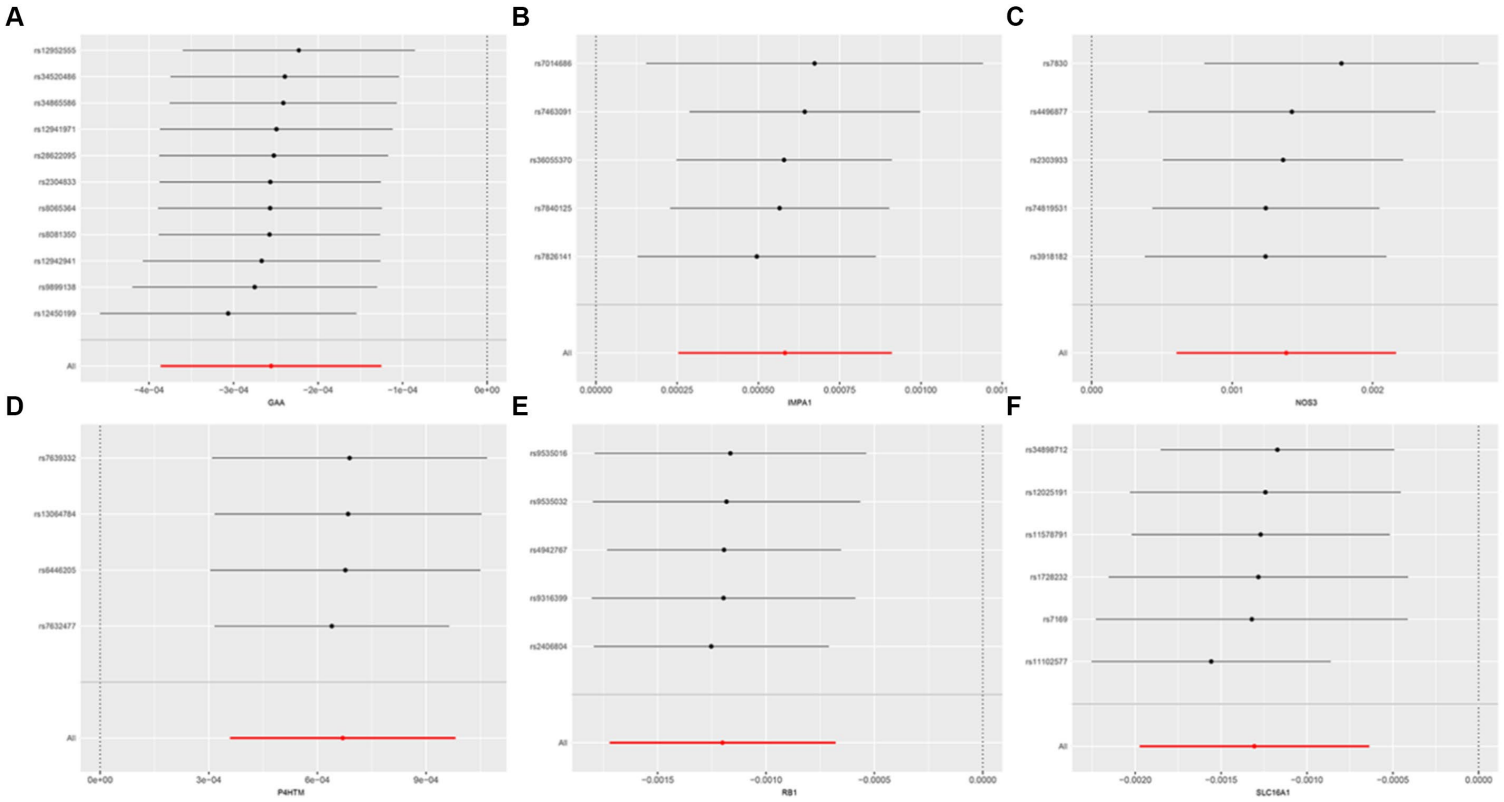
Sensitivity analysis of druggable genes on secondary malaise and fatigue. **(A)** GAA, **(B)** IMPA1, **(C)** NOS3, **(D)** P4HTM, **(E)** RB1, **(F)** SLC16A1.

The study examined the MR correlations between 1,450 proteins that have accessible index eQTL signals and the risk of SMF outcomes. The results of this investigation may be seen in the Supplementary material. We found 176 protein-SMF pairings with marginal significance (*p* < 0.05). After removing and integrating false positive and repetition targets, 48 prospective RSYRD targets against SMF were found. After MR quality control it was shown that 37 candidate druggable genes have the most robust MR evidence for SMF risk (Figure 2). Figure 3 depicts an overview of the MR analysis results, which identify 17 prospective druggable genes for SMF after FDR adjustment (FDR <0.05) (Supplementary Table S1). Ultimately, NOS3, GAA, IMPA1, P4HTM, RB1, and SLC16A1, as potential targets of RSYRD for SMF reached FDR <0.05 (Figure 4A). Genetic predictions indicate that elevated levels of IMPA1, P4HTM, and NOS3 are linked to a higher risk of SMF. Conversely, lower levels of GAA, RB1, and SLC16A1 are associated with an increased risk of SMF. This suggests that the three proteins are negatively correlated with SMF risk (Figure 4B). In addition, the sensitivity analysis indicated that there was no variation or cross-effects in any of the other outcomes (*p* > 0.05). The leave-one-out technique revealed that eliminating any SNP for SMF made no change in the results (Figure 5).

### 3.3 H-I-T network construction and topological network analysis

**Figure 6:**
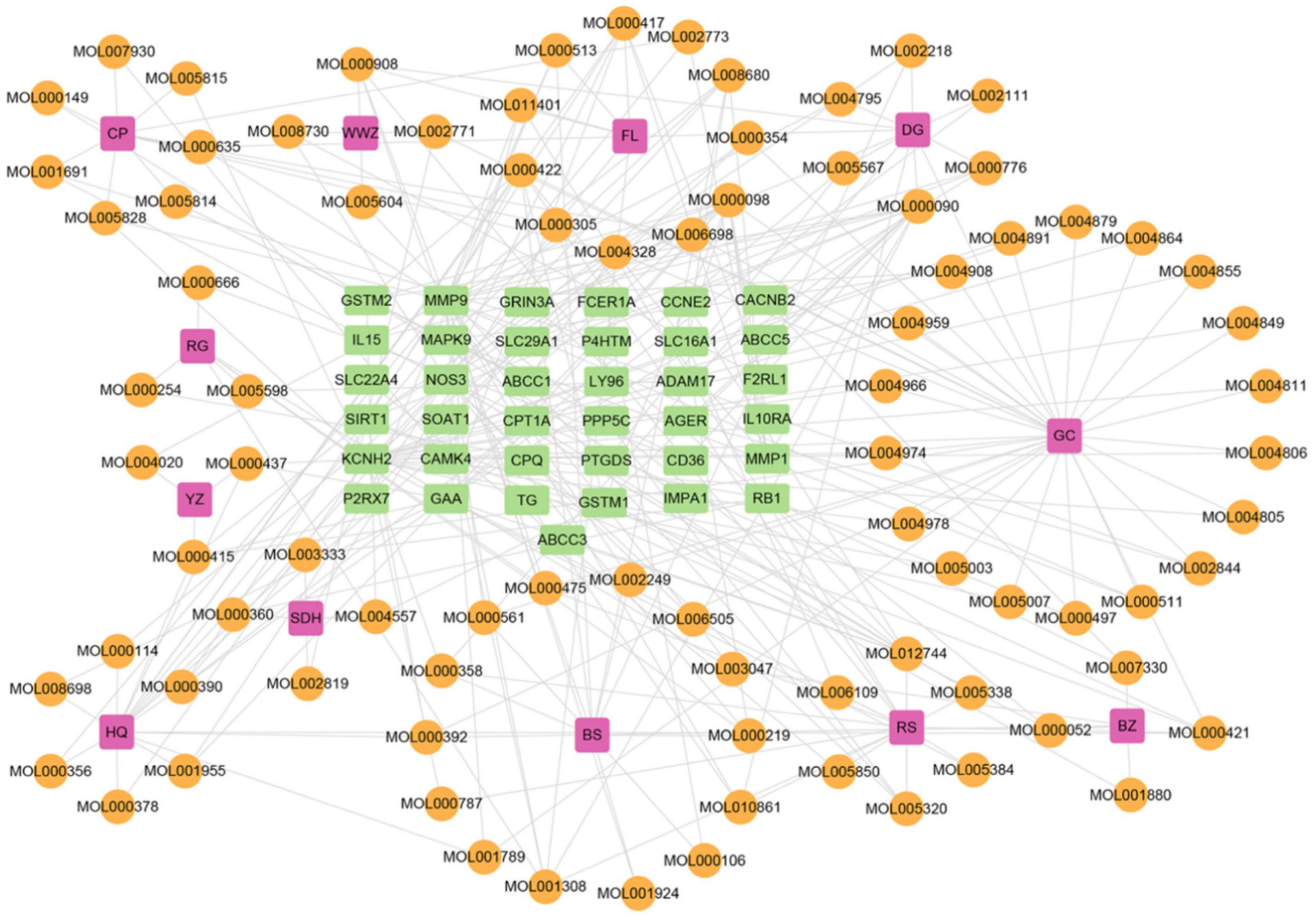
The network of herb-ingredient-target includes 12 kinds of herbs, 86 active ingredients, and 37 target genes.

**Figure 7:**
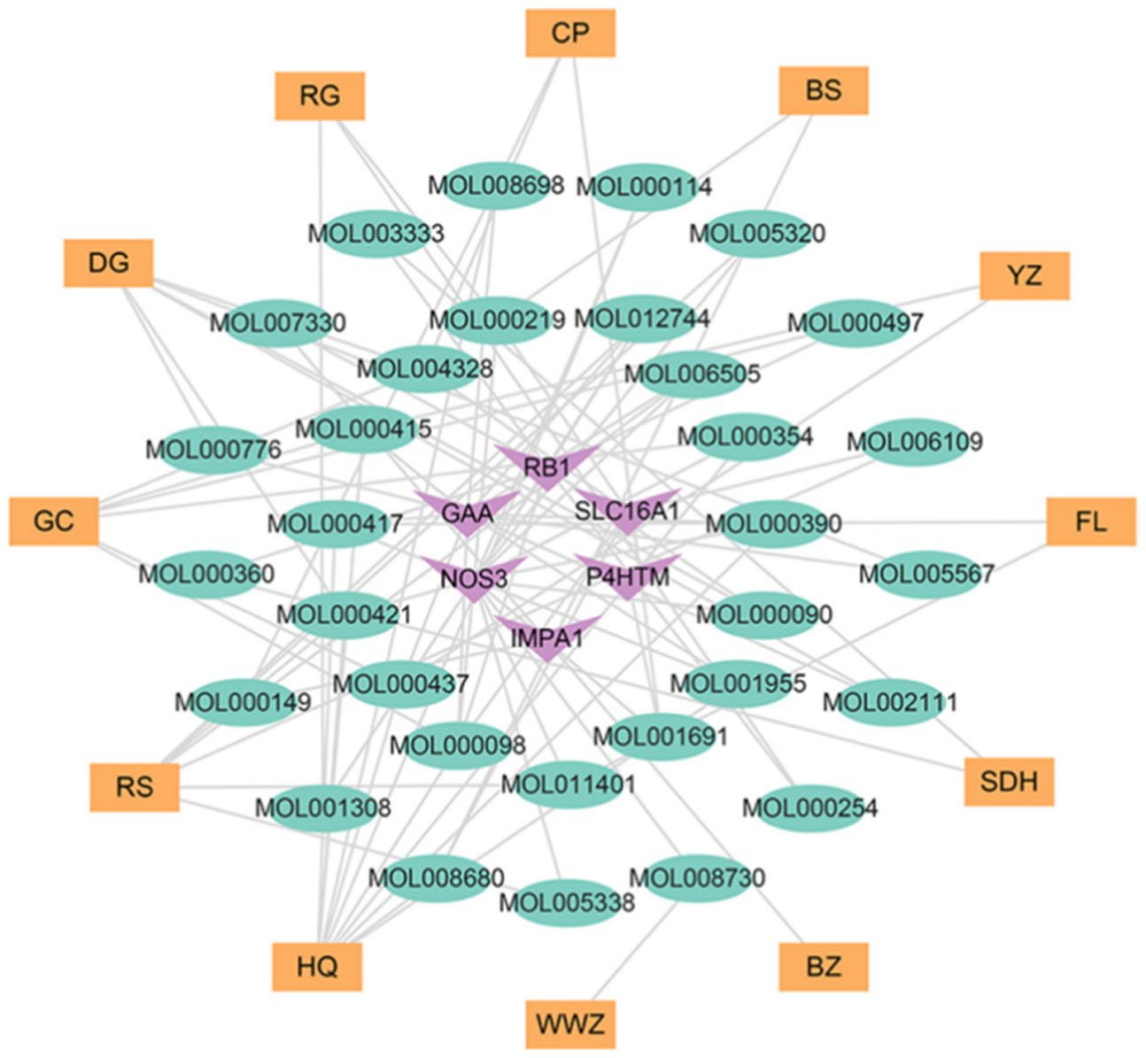
The network of herb-ingredient-target includes 12 kinds of herbs, 32 active ingredients, and 6 target genes.

On a system level, the H-I-T network with 135 nodes and 282 edges was built by Cytoscape 3.9.1 (Figure 6) to gain a better knowledge of the interaction between 12 herbs, 86 ingredients, and 37 intersection target genes. To assess node degree values, we used Cytoscape’s built-in NetworkAnalyzer. Among these, the active components in Gancao with the greatest number of associated targets were Quercetin (degree = 14), Kaempferol (degree = 8), and Naringenin (degree = 6), and the active ingredients with more targets in Huangqi, Renshen, and Danggui were Curcumin (degree = 13), Resveratrol (degree = 9), Capsaicin (degree = 7), Acetaldehyde (degree = 6), BETA-ELEMENE (degree = 6) and Oleic Acid (degree = 6). Furthermore, the targets connecting more active ingredients included matrix metalloproteinase-9 (MMP9) (degree = 25), potassium voltage-gated channel subfamily H member 2 (KCNH2) (degree = 21), nitric oxide synthase 3 (NOS3) (degree = 17), alpha glucosidase (GAA) (degree = 12) and sirtuin 1 (SIRT1) (degree = 10). Further analysis shows that the herb-ingredient-target network comprised a total of 50 nodes (12 herb nodes, 32 ingredient nodes, 6 target nodes) and 91 edges (Figure 7). Huangqi (degree = 10) and Renshen (degree = 7) are the main effective ingredients. Table 1 lists the effective components and indications of crude herbs in RSYRD for the treatment of SMF (53–61, 63–66).

**Table 1:** Effective components and indications of crude herbs in RSYRD.

Crude herb	Effective component	Indication of crude herb
Renshen	Ginsenoside Re, Chikusetsusaponin IVc, Arachidonic Acid, Nicotinic Acid, Pyruvic Acid, Resveratrol, (−)-Epicatechin	Anti-aging, anti-diabetic, immunoregulatory, anti-cancer, neuroregulation, wound and ulcer healing activity (53)
Baizhu	L-Menthol	Immunomodulatory, antitumour, gastroprotective and intestinal health-promoting, hepatoprotective, hypoglycaemic as well as other activities (54)
Chenpi	L-Ascorbic Acid, Naringenin, Inositol	Antioxidant, anti-inflammatory, antibacterial properties, and anti-cancer activity, hypolipidemic, antiplatelet activity (55, 56)
Gancao	Licochalcone A, Isoquercitrin, Rutin, Isorhamnetin, Naringenin, Quercetin	Adrenocortical hormone-like effects, anti-inflammatory, anti-cancer and immunomodulatory effects (57)
Fuling	Capsaicin, Ginsenoside Re	Anti-tumor, anti-bacterial, anti-oxidant, anti-inflammatory, immunomodulation, and liver and kidney protection (58)
Huangqi	Chlorogenic Acid, Daidzein, Dihydrocapsaicin, Nicotinic Acid, Acetaldehyde, Capsaicin, Quercetin, Isoquercitrin, Isorhamnetin, Rutin	Antioxidant, anti-inflammatory, immunoregulatory, anticancer, hypolipidemic, antihyperglycemic, hepatoprotective, expectorant, and diuretic effects (59)
Baishao	Acetaldehyde, Benzoic acid	Anti-inflammatory and immunomodulatory effects, stimulate blood circulation and exhibit, antiplatelet, and vasodilator activities (60)
Danggui	Nicotinic Acid, Naringenin, Ethanol, Curcumin, (Z)-Butylidenephthalide, Retinol	Inhibition of inflammatory factor release, anti-oxidative injury, and interference with collagen production (61)
Shudihuang	Ferulic acid, Acteoside	Analgesia, sedation, anti-tumor, anti-inflammation, antioxidation, immunomodulation, cardiovascular and cerebrovascular regulation, and nerve damage repair (62)
Wuweizi	Nordihydroguaiaretic acid	Antioxidants, anti-inflammatory, antiviral, anticancer, and anti-aging effects (63)
Rougui	Benzoic acid, Oleic acid, Eugenol	Antioxidant, antitumor, analgesics, antihypertension, anticoagulant, anti-inflammatory, antidiabetic effects (64)
Yuanzhi	Isoquercitrin, Rutin	Antioxidation, anti-inflammation, antidementia, and anti-aging (65)

### 3.4 GO and KEGG pathway enrichment analyses

**Figure 8:**
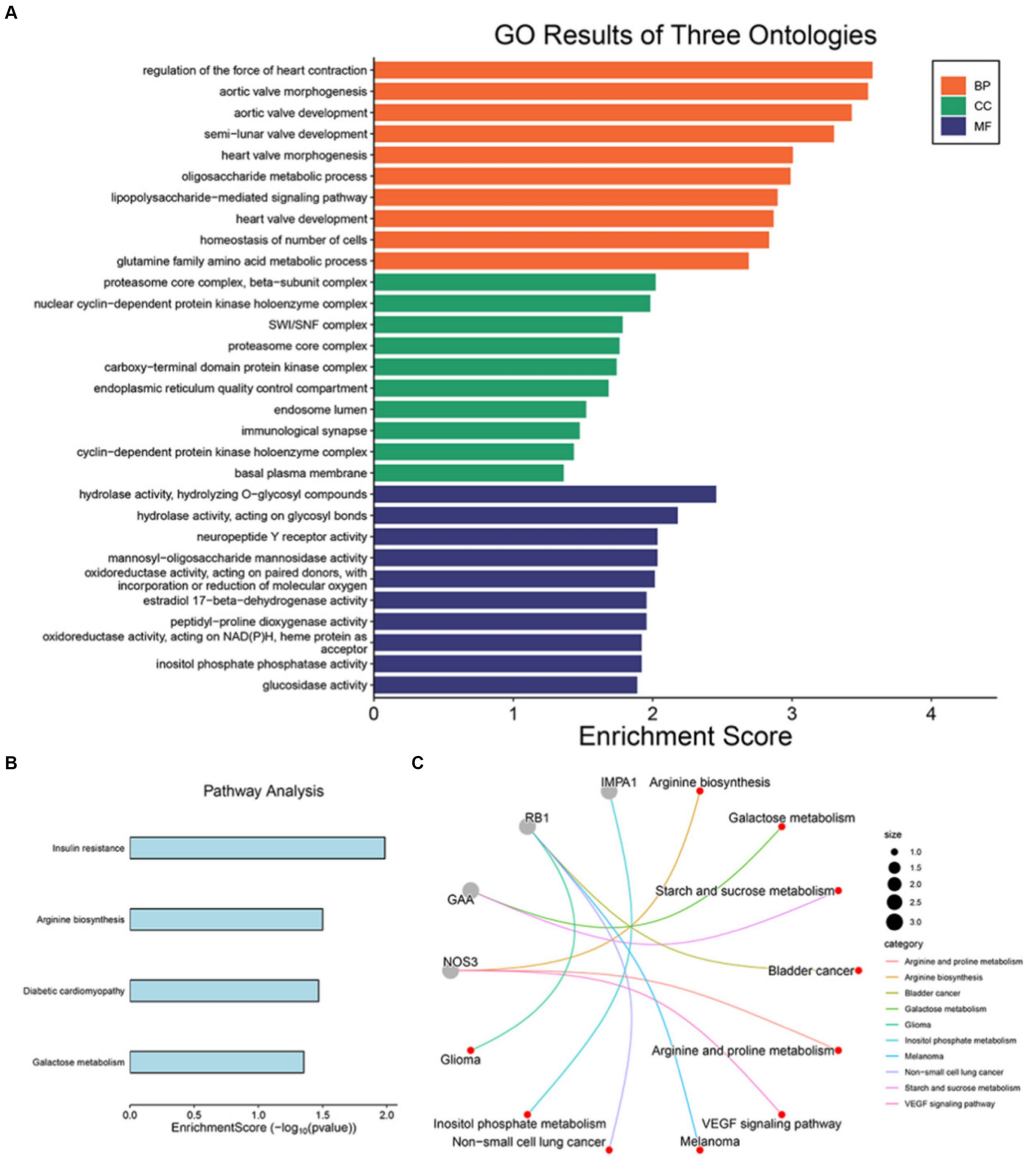
GO and KEGG pathway enrichment. **(A)** GO and **(B)** KEGG pathway enrichment of 17 druggable genes of SMF. **(C)** KEGG pathway enrichment of 6 prospective therapeutic target genes.

We conducted biological pathway enrichment analysis of 17 druggable genes obtained through Mendelian Randomization to explore the prospective therapeutic target genes of SMF, and further analyzed NOS3, GAA, IMPA1, P4HTM, RB1, and SLC16A1 to clarify the potential mechanism of RSYRD in treating SMF. As shown in Figure 8A, the most significant pathways in the BP category were all associated with regulation of the force of heart contraction, oligosaccharide metabolic process, lipopolysaccharide-mediated signaling pathway, homeostasis of number of cells, and glutamine family amino acid metabolic process. In class CC, drug target genes are similarly enriched for endoplasmic reticulum-related components (immunological synapse, SWI/SNF complex, endoplasmic reticulum quality control compartment, and endosome lumen), which is consistent with previous studies (67). Furthermore, in terms of MF, these genes are also involved in functions strongly associated with energy metabolism (hydrolase activity, mannosyl-oligosaccharide mannosidase activity, and oxidoreductase activity, acting on NAD (P) H, heme protein as acceptor). As shown in Figure 8B, the four pathways analysed by KEGG enrichment are insulin resistance, arginine biosynthesis, diabetic cardiomyopathy and galactose metabolism, all four of which are closely linked to the metabolic syndrome. Similarly, NOS3, GAA, IMPA1, P4HTM, RB1, and SLC16A1 are associated with metabolic pathways such as arginine and proline metabolism, galactose metabolism, starch and sucrose metabolism, and inositol phosphate metabolism (Figure 8C) (68).

### 3.5 PPI and druggability prediction on therapeutic target’s potential

The protein–protein interaction analysis discovered just one interaction between the identified potential causative proteins, P4HTM and GAA, which were engaged in signal transduction and glucose metabolism pathways, respectively. The present research utilized the DSigDB database to predict potentially useful interventional drugs. The following proteins (NOS3, GAA, IMPA1, P4HTM, RB1, and SLC16A1) were targeted for drug development, according to our evaluation of druggability. We used Enrichr to identify possible drug molecules based on transcriptome characterization in the DSigDB database. After taking the intersection with the active ingredients of RSYRD, 14 potential drugs were recommended to act on target genes based on adjusted *p*-value (Table 2). This indicates that the active ingredients of RSYRD can be used for the treatment of SMF.

**Table 2:** Candidate drug predicted using DSigDB.

Term	*p*-value	Adjusted *p*-value	Genes
Benzoic acid CTD 00007316	0.001	0.003	SLC16A1; NOS3
Oleic acid CTD 00007269	0.000	0.005	RB1; NOS3
Pyruvic acid CTD 00007265	0.003	0.027	SLC16A1
Rutin TTD 00010730	0.004	0.027	GAA
Isoquercitrin TTD 00008703	0.004	0.027	GAA
(−)-Epicatechin TTD 00000019	0.004	0.027	GAA
Ginsenoside Re CTD 00002168	0.005	0.027	NOS3
Isorhamnetin CTD 00002092	0.005	0.027	NOS3
Ferulic acid CTD 00000186	0.005	0.027	RB1
Quercetin CTD 00006679	0.007	0.027	RB1; SLC16A1; NOS3; GAA
Curcumin CTD 00000663	0.010	0.030	RB1; NOS3
Chlorogenic acid CTD 00005640	0.012	0.030	NOS3
Naringenin CTD 00000211	0.013	0.030	SLC16A1
Acteoside CTD 00002463	0.013	0.030	RB1

### 3.6 Validation of the relationship between RSYRD ingredients and core targets by molecular docking

**Figure 9:**
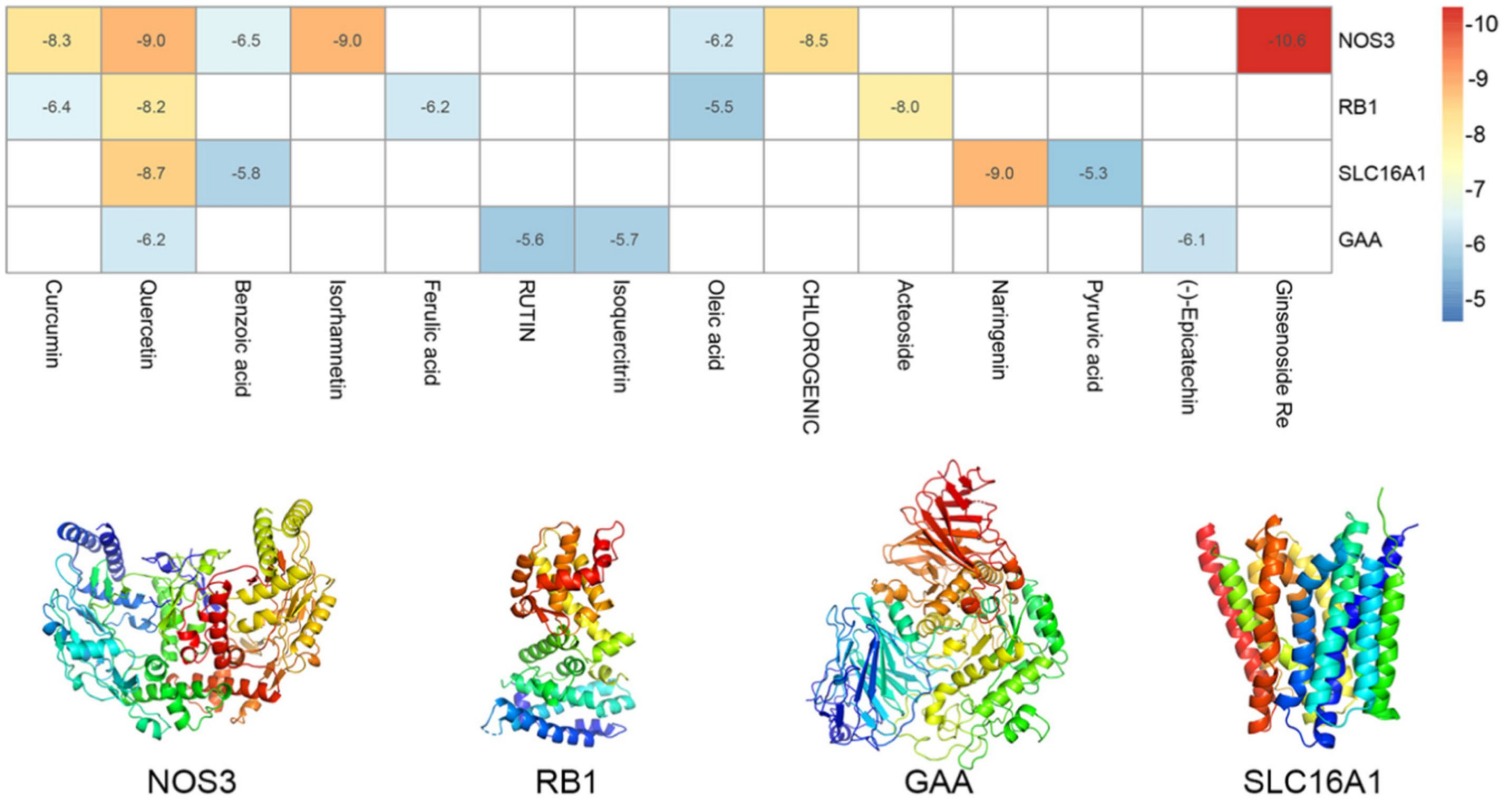
Heatmaps of the docking scores of target genes combined with active ingredients of RSYRD. The 3D structure of target genes was displayed.

**Figure 10:**
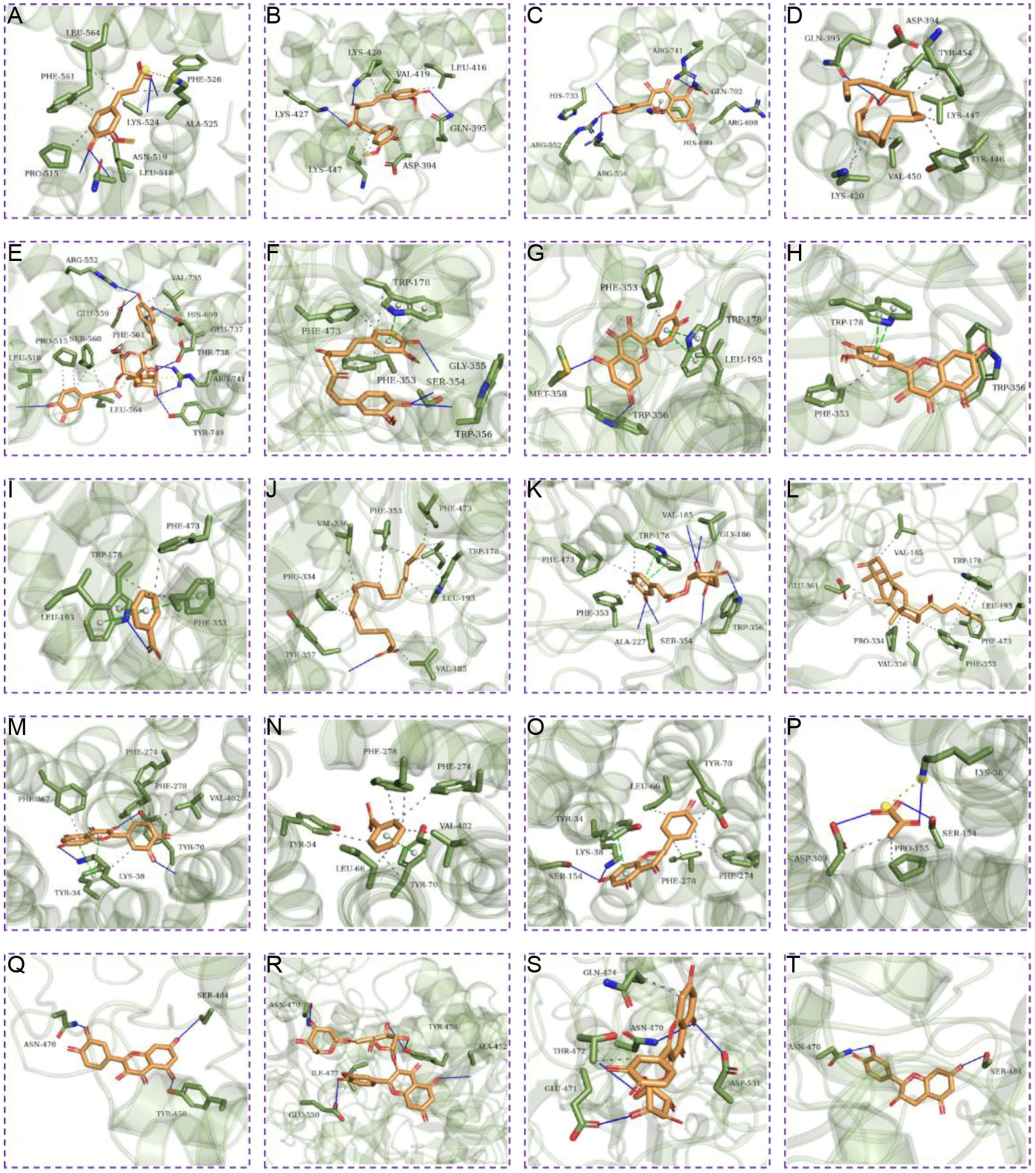
Molecular docking of target genes with active ingredients of RSYRD. **(A)** Ferulic acid docking RB1. **(B)** Curcumin docking RB1. **(C)** Quercetin docking RB1. **(D)** Oleic acid docking RB1. **(E)** Acteoside docking RB1. **(F)** Curcumin docking NOS3. **(G)** Quercetin docking NOS3. **(H)** Isorhamnetin docking NOS3. **(I)** Benzoic acid docking NOS3. **(J)** Oleic acid docking NOS3. **(K)** Chlorogenic acid docking NOS3. **(L)** Ginsenoside Re docking NOS3. **(M)** Quercetin docking SLC16A1. **(N)** Benzoic acid docking SLC16A1. **(O)** Naringenin docking SLC16A1. **(P)** Pyruvic acid docking SLC16A1. **(Q)** Quercetin docking GAA. **(R)** Rutin docking GAA. **(S)** Isoquercitrin docking GAA. **(T)** (−)-Epicatechin docking GAA.

Further molecular docking simulations were performed to further investigate the interactions between the active ingredients of RSYRD and core targets (NOS3, GAA, RB1, and SLC16A1) and to elucidate the Traditional Chinese medicine formulas development plan. According to the molecular docking data, the binding energy among the core target proteins and the effective chemically active ingredients was below −5 kcal/mol, indicating that the binding activity among the core target proteins and the functional core ingredients was stable (Figure 9). Figure 10 shows the docking results of the active ingredients with the best binding affinity for each core target protein. Taken together, these RSYRD representative compounds may bind well to the aforementioned core SMF targets, all of which may play important roles in SMF treatment.

### 3.7 Identification of DEGs and clinical characteristics of key targets

**Figure 11:**
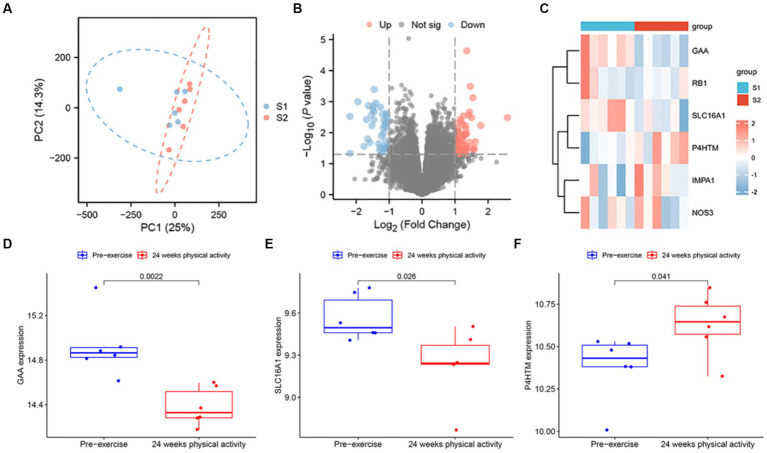
SMF mRNA Expression analysis using GEO Dataset. **(A)** Principal component analysis for GSE12385. **(B)** Volcano of the differentially expressed genes. The red dots represent the significantly up-regulated genes and the blue suggest the significantly down-regulated genes. **(C)** Heatmap of the 6 key genes in blood samples at 2 time points. The box plot of the GAA **(D)**, SLC16A1 **(E)**, and P4HTM **(F)** in the GSE12385 dataset.

To evaluate the intra-group data repeatability, we performed principal component analysis (PCA), and the results showed that the repeatability of data in GSE12385 is fine (Figure 11A). Subsequently, gene expression changes in Peripheral Blood Mononuclear cells (PBMC) induced by physical activity was investigated in sedentary middle-aged men who undertook a 24-weeks physical activity programme, and to evaluate the expression changes of 6 key targets in blood sampling. Following the analysis of the GSE12385 dataset with R software, the differentially expressed genes between the pre-exercise period and at the end of 24-weeks prescribed physical activity were presented in volcano plot (Figure 11B). Heatmap displays the expression of six key genes (Figure 11C). Moreover, box plots showed the expression patterns of 3 key genes (Figures 11D−F). The results showed a significant increase in mRNA expression of GAA and SLC16A1 in blood sampling among baseline males. On the contrary, P4HTM was significantly upregulated at the end of the prescribed 24 weeks physical activity.

## 4 Discussion

In order to explore and understand the therapeutic mechanisms of Traditional Chinese Medicine, we establish a framework that can connect classical Chinese medicine theory to modern biomedical science and can handle the complexity of herbs’ chemical composition and therapeutic protein target. Additionally, this is the initial research to use Mendelian randomization in the study along with network pharmacology. First, network pharmacology was used to examine the active components and protein targets of RSYRD for SMF therapy. We can analyze the “herb-ingredient-proteins/genes-disease” interacting network through the perspective of systems biology using biological databases as well as clinical trial findings, which will offer knowledge of how the actions of herbs/TCM recipes treat disease and ZHENG. Furthermore, we employ the Mendelian randomization technique along with extensive genome-wide association research data to identify novel therapeutic targets (NOS3, GAA, IMPA1, P4HTM, RB1, and SLC16A1) for SMF. This approach enhances the efficacy of treatment prediction by demonstrating a cause-and-effect relationship between exposure and result by the utilization of cis-eQTL and SMF risk association data. Finally, to demonstrate the druggability of these target genes, we predicted and molecular docked the medicines corresponding to these targets.

According to the results achieved here, four protein targets in SMF and 14 active ingredients in RSYRD were identified, suggesting that RSYRD exerted its pharmacological effects on treating SMF through multi-ingredients and multi-targets. RSYRD is a formula composed of 12 different herbs, with Huangqi and Renshen forming its most effective ingredients. In traditional Chinese medicine, Huangqi is frequently used for treating patients who have Qi deficiency as well as has been demonstrated to have a strong antifatigue effect (69–71). Renshen was a potential treatment for exhaustion in chronic illness, with few documented side effects (72). Nonetheless, there is little data to support the effectiveness of ginseng supplements in reducing fatigue and boosting athletic performance (73–76). Previous studies have identified polysaccharides, polyphenols, flavonoids, terpenes, peptides, and other components extracted from Chinese herbal medicine as potential anti-fatigue agents (77), quercetin (78), curcumin (79), ginsenoside (80), rutin (81), chlorogenic acid, ferulic acid, isoquercitrin, and other natural products have been shown to exhibit definite anti-fatigue properties (77). Nevertheless, Chinese medical formulae are not a patchwork of drugs with the same properties, and there are strict principles for making prescriptions. The reasons for this are that the action of a single drug is usually limited, and some of them might cause adverse effects or even toxicity. However, when numerous medications are used together, ensuring that their advantages are fully utilized while limiting their disadvantages, they will demonstrate their superiority over a single drug in the treatment of diseases (82). Single-target intervention has been shown to be ineffective and insufficient in complex diseases with robust biological networks, such as cancer (39). In these circumstances, Chinese medicinal formulas techniques can simultaneously target numerous disease targets, resulting in chemical-protein interaction (83, 84).

In this study, SLC16A1, GAA, NOS3, and RB1 were determined to be the four hub protein targets associated with SMF. Solute carrier family 16 member 1 (SLC16A1; also known as MCT1) encodes a transmembrane protein that enables proton-linked transport of a variety of monocarboxylate metabolites across the cellular membrane, including lactate, pyruvate, and ketone bodies (85–87). Lactate is a significant circulating carbohydrate fuel (88, 89). MCTs 1–4 (Slc16a1, Slc16a7, Slc16a3, and Slc16a4) regulate lactate entrance and departure from cells. These proteins’ expression and activity may be modulated to affect lactate location in the body (90). Based on the existence of lactate shuttles, it is postulated that MCT1 expression may be significant for blood lactate elimination following supramaximal exercise, resulting in a better tolerance to muscle exhaustion (91, 92). GAA encodes the lysosomal enzyme acid alpha-glucosidase, which causes lysosomal glycogen buildup, swelling, and rupture in all tissues of the human body. Furthermore, autophagic accumulation, organelle abnormalities, and energy deficiencies are common (93). Pompe illness is characterized by a partial or total GAA deficiency, and in addition to symptoms associated with skeletal and respiratory muscle weakness, non-motor issues such as weariness can have a dramatic and devastating impact on the patient’s life (94). Aval glucosidase alfa gained its initial approval in the United States in August 2021 for the treatment of patients 1 year of age and older with late-onset Pompe disease (GAA deficiency) (95, 96). The NOS3 gene has several genetic variations, including single nucleotide polymorphisms (SNPs), variable number of tandem repeats (VNTRs), microsatellites, and insertions/deletions (97). Endothelial nitric oxide synthase (NOS3) is an essential enzyme responsible for the synthesis of nitric oxide (NO) in the endothelial cells lining the blood vessels (98). Under normal physiological settings, NO is produced in cells by the enzyme nitric oxide synthase (NOS) converting l-arginine to l-citrulline (99). Nitric oxide regulates various physiological processes, such as muscle fiber type, microtubule organization, fatigability, postexercise force recovery, and mitochondrial ATP synthesis efficiency, through cGMP-dependent mechanisms (100, 101). The Retinoblastoma Susceptibility gene (RB1) was the initial human tumor suppressor gene to be discovered, and it plays a crucial role in the formation of retinoblastoma, a type of cancer that affects children’s eyes (102). It could be employed as a biomarker for breast cancer cell sensitivity to GLUT1 inhibitors (103). These findings indicate that the RSYRD medication targets presented in this work are significantly linked to SMF and have a high medicinal potential, suggesting that Chinese medicine formulas targeting these genes for SMF could be developed.

The current study provides several notable advantages. First, this is the first study to employ MR to identify SMF therapeutic targets, based on data from the biggest publicly available SMF risk GWAS to date. Furthermore, network pharmacology, which is based on a “herb-ingredient-target” network, can expose small-molecule regulation principles in a high-throughput way and illuminate the complicated interaction between pharmacological targets and the material basis of RSYRD. The conclusive drug predictions showcase the medical potential of these genes, while the strong binding activity seen in molecular docking highlights their substantial value as drug targets. This study offers a thorough assessment, covering everything from identification to drug-binding qualities, and it provides solid evidence for four potential therapeutic targets for SMF. The method unveils the scientific foundation of TCM and creates a paradigm for understanding the molecular basis of Chinese medical formulas and predicting disease treatments. Additionally, additional research on RSYRD may have a significant impact on the development of novel targeted therapeutics for SMF, perhaps by the isolation and characterisation of the active chemicals in the efficacious Chinese medical formulae.

## 5 Limitations

Several limitations must be considered when evaluating this study. It is important to note that certain medications and target genes may not have been incorporated into the public database, mostly because of the inherent characteristics of network pharmacology research. The formulation of Chinese herbal medicine is intricate, and it remains uncertain whether there are synergistic benefits or negative effects among substances that are difficult to elucidate. Another limitation arises from the diverse composition of the study sample. Although the investigation of eQTLs includes individuals from non-European backgrounds, the SMF population consists exclusively of individuals of European descent. Due to differences in genetic background and patterns of linkage disequilibrium, variations in demographic backgrounds may introduce potential bias in calculations of the influence of Mendelian randomization. The study’s generalizability is constrained by its overrepresentation of individuals of European descent. Further investigation and verification are required to extend the results to individuals of different ethnic backgrounds, in order to ensure the wider applicability of the findings. The accuracy of molecular docking analysis is greatly influenced by the quality of the protein structures and ligands. Although this strategy identifies potential drug targets, it does not guarantee their effectiveness in real-world clinical scenarios. Additional experimental validation and clinical trials are necessary to determine the therapeutic potential of the identified targets.

## 6 Conclusion

In conclusion, MR analysis was used in this study to find potential drug targets for secondary malaise and fatigue. To discover the active components and anti-SMF targets of RSYRD, we employed the network pharmacology approach to generate a multi-dimensional network map. Our comprehensive study indicates a causal relationship between the risk of SMF and genetically inherited levels of circulating NOS3, GAA, IMPA1, P4HTM, RB1, and SLC16A1. The identified proteins may be appealing drug targets for SMF, especially NOS3, GAA, and SLC16A1. Furthermore, the medicinal efficacy of these targets was verified through the application of molecular docking and drug prediction. The results provide encouraging avenues for more potent SMF therapies, which could lower the cost of medication research and advance personalised medicine strategies.

## Data availability statement

The original contributions presented in the study are included in the article/Supplementary material, further inquiries can be directed to the corresponding authors.

## Author contributions

FW: Conceptualization, Data curation, Formal analysis, Funding acquisition, Investigation, Methodology, Project administration, Resources, Software, Supervision, Validation, Visualization, Writing – original draft. LZ: Conceptualization, Data curation, Formal analysis, Investigation, Methodology, Project administration, Resources, Software, Supervision, Validation, Visualization, Writing – original draft. HC: Conceptualization, Data curation, Formal analysis, Investigation, Methodology, Project administration, Resources, Software, Supervision, Validation, Visualization, Writing – original draft. SG: Writing – review & editing. JW: Writing – review & editing. AL: Conceptualization, Data curation, Formal analysis, Investigation, Methodology, Project administration, Resources, Software, Supervision, Validation, Visualization, Writing – original draft. ZW: Conceptualization, Data curation, Formal analysis, Funding acquisition, Investigation, Methodology, Project administration, Resources, Software, Supervision, Validation, Visualization, Writing – original draft.

## Footnotes

^1^ http://mpns.kew.org

^2^ http://tcmspw.com/tcmsp.php

^3^ http://bionet.ncpsb.org/batman-tcm/

^4^ https://sparql.uniprot.org/

^5^ https://eqtlgen.org/

^6^ https://gwas.mrcieu.ac.uk/datasets/ukb-b-11972/

^7^ https://string-db.org/

^8^ http://dsigdb.tanlab.org/DSigDBv1.0/

^9^ https://amp.pharm.mssm.edu/Enrichr/

^10^ https://pubchem.ncbi.nlm.nih.gov/

^11^ https://www.rcsb.org/

^12^ http://www.ncbi.nlm.nih.gov/geo/
